# An Interactive Voice Response and Text Message Intervention to Improve Blood Pressure Control Among Individuals With Hypertension Receiving Care at an Urban Indian Health Organization: Protocol and Baseline Characteristics of a Pragmatic Randomized Controlled Trial

**DOI:** 10.2196/11794

**Published:** 2019-04-02

**Authors:** Emily B Schroeder, Kelly Moore, Spero M Manson, Megan A Baldwin, Glenn K Goodrich, Allen S Malone, Lisa E Pieper, Stanley Xu, Meredith M Fort, David Johnson, Linda Son-Stone, John F Steiner

**Affiliations:** 1 Kaiser Permanente Colorado Institute for Health Research Aurora, CO United States; 2 Department of Medicine University of Colorado Anschutz Medical Campus Aurora, CO United States; 3 Centers for American Indian and Alaska Native Health University of Colorado Anschutz Medical Campus Aurora, CO United States; 4 First Nations Community HealthSource Albuquerque, NM United States

**Keywords:** Indians, North American, hypertension, urban health, pragmatic clinical trial, text messages

## Abstract

**Background:**

Efficient and effective strategies for treating chronic health conditions such as hypertension are particularly needed for under-resourced clinics such as Urban Indian Health Organizations (UIHOs).

**Objective:**

The objective of the Controlling Blood Pressure Trial is to assess the impact of an interactive voice response and text message (IVR-T) intervention compared with usual care among individuals with hypertension receiving care at a UIHO in Albuquerque, New Mexico. This manuscript presents the baseline characteristics of individuals enrolled in the trial and compares their characteristics with those in the hypertension registry who did not enroll in the trial.

**Methods:**

A hypertension registry developed from the clinic’s electronic health record was used for recruitment. Potentially eligible participants were contacted by letter and then by phone. Those who expressed interest completed an in-person baseline visit that included a baseline survey and blood pressure measurement using standardized procedures. Individuals randomized to the intervention group could opt to receive either automated text messages or automated phone calls in either English or Spanish. The messages include reminders of upcoming appointments at First Nations Community HealthSource, requests to reschedule recently missed appointments, monthly reminders to refill medications, and weekly motivational messages to encourage self-care, appointment keeping, and medication taking for hypertension. Individuals in the IVR-T arm could opt to nominate a care partner to also receive notices of upcoming and missed appointments. Individuals in the IVR-T arm were also offered a home blood pressure monitor. Follow-up visits will be conducted at 6 months and 12 months.

**Results:**

Over a 9.5-month period from April 2017 to January 2018, 295 participants were enrolled from a recruitment list of 1497 individuals. The enrolled cohort had a mean age of 53 years, was 25.1% (74/295) American Indian or Alaska Native and 51.9% (153/295) Hispanic, and 39.0% (115/295) had a baseline blood pressure greater than or equal to 140/90 mmHg. Overall, the differences between those enrolled in the trial and patients with hypertension who were ineligible, those who could not be reached, or those who chose not to enroll were minimal. Enrolled individuals had a slightly lower blood pressure (129/77 mmHg vs 132/79 mmHg; *P=*.04 for systolic blood pressure and *P*=.01 for diastolic blood pressure), were more likely to self-pay for their care (26% vs 10%; *P*<.001), and had a more recent primary care visit (164 days vs 231 days; *P*<.001). The enrolled cohort reported a high prevalence of poor health, low socioeconomic status, and high levels of basic material needs.

**Conclusions:**

The Controlling Blood Pressure Trial has successfully enrolled a representative sample of individuals receiving health care at a UIHO. Trial follow-up will conclude in February 2019.

**Trial Registration:**

ClinicalTrials.gov NCT03135405; http://clinicaltrials.gov/ct2/show/NCT03135405 (Archived by WebCite http://www.webcitation.org/76H2B4SO6)

**International Registered Report Identifier (IRRID):**

DERR1-10.2196/11794

## Introduction

### Background

American Indians and Alaska Natives (AI/ANs) face pervasive health disparities in comparison with other racial and ethnic groups in the United States. Although disparities in diabetes have received national attention, cardiovascular disease (CVD) remains the primary cause of mortality in AI/ANs as in other groups [1]. Interventions to prevent CVD in AI/ANs have historically focused on individuals with diabetes (eg, the Special Diabetes Program for Indians Healthy Heart Demonstration Project) [2], with few studies of interventions to prevent CVD among AI/ANs without diabetes. Successful CVD prevention for AI/ANs requires the engagement of health care systems dedicated to this population. The health care system for AI/ANs includes federally operated Indian Health Service (IHS) facilities, tribally operated facilities, and 33 Urban Indian Health Organizations (UIHOs) across the United States. All face unremitting challenges in funding and staffing that jeopardize their mission to provide culturally appropriate health care, outreach, and referral services for AI/AN. Strategies for treating chronic health conditions are particularly problematic in UIHOs. Although 71% of AI/ANs live in urban areas, UIHOs only receive 1% of the IHS budget [3,4].

Treatment of hypertension is a pillar of CVD prevention. Hypertension is the most common chronic health condition in the United States [5]. Uncontrolled hypertension increases the risk of myocardial infarction, stroke, kidney failure, and congestive heart failure [6]. Hypertension rates are high, levels of control are low, and disparities in care are evident in AI/AN populations [1,7-9]. Communication technologies that extend care to patients “where they are,” outside the clinical setting, can facilitate hypertension control [10]. Such outreach may be particularly important for AI/ANs and other vulnerable populations that face logistical and cost barriers to receiving office-based care.

Interactive Voice Response and Text Message (IVR-T) technology can be used to send targeted voice messages to landlines and voice or text messages to cell phones. IVR-T tools could improve hypertension care by sending reminders about appointments or medication refills, recalling individuals who have missed appointments, and providing educational and motivational messages [11]. Existing evidence suggests that such interventions can improve medication adherence [12-15], appointment keeping [16], and blood pressure control [14,17]. Randomized controlled trials (RCTs) of IVR-T interventions have demonstrated clinical benefit at a low marginal cost [18-21]. In resource-constrained UIHOs, IVR-T messages may substitute for costly and time-intensive personal outreach by staff members. In addition, home blood monitoring without additional support has been shown to have a modest effect of systolic blood pressure at 6 months, but little sustained effect [22,23].

### Objectives

The specific aim of the Controlling Blood Pressure Trial is to assess the impact of an IVR-T intervention compared with usual care among individuals with hypertension receiving care at a UIHO in Albuquerque, New Mexico. The primary study endpoint is change in mean systolic blood pressure between baseline and 12 months. Secondary endpoints include change in diastolic blood pressure, self-reported adherence, and the proportion of missed clinic appointments between intervention and control groups.

## Methods

### Study Setting

This pragmatic clinical trial is taking place at First Nations Community HealthSource (FNCH), a nonprofit, urban Indian community-based health and human services organization incorporated in 1972. The health center is authorized through Title V of P.L. 94-437, and subsequent amendments, of the Indian Health Care Improvement Act to improve the health of urban Indians. FNCH has expanded its mission to serve the greater community, without losing its fundamental basis for existence, and now provides primary medical care to urban AI/ANs and other socially disadvantaged residents of the Albuquerque, New Mexico area. In 1997, the center was designated as a Federally Qualified Health Center, and it is an Accreditation Association for Ambulatory Health Care (AAAHC)-Accredited Health Center and Medical Home. Approximately 40% of FNCH clients are AI/AN; Diné (Navajo) is the most common tribal affiliation. Many other clients served by FNCH are undocumented immigrants. Housing insecurity and homelessness are common. Most clients speak either English or Spanish as their primary language. FNCH has 3 campuses in close geographic proximity in southeastern Albuquerque. The medical clinic is staffed by physicians, nurse practitioners, and physician assistants with extensive support staff including public health nurses, diabetes educators, patient navigators, and on-site behavioral health clinicians. FNCH provides comprehensive primary medical, dental, behavioral health, and traditional healing and a range of preventive support services to address the health disparities experienced by its target population. Its mission is to provide a culturally competent, comprehensive health service delivery system integrating traditional values to enhance the physical, spiritual, emotional, and mental well-being of American Indian families and other underserved populations residing in Albuquerque and on tribal reservations.

The study team established an Advisory Council consisting of FNCH staff from pharmacy, public health nursing, and social services and 2 patients and their caregivers. The Advisory Council actively collaborated in designing the trial, including participant eligibility criteria, recruitment materials, incentives for participation, operationalization of the intervention, participant survey materials, and the timing and content of the IVR-T messages. The Advisory Council met 3 times before starting the study and has met at least twice annually during the study itself.

### Eligibility and Recruitment

Consistent with principles of a pragmatic trial, study inclusion criteria are broad, to demonstrate the impact of the intervention in a “real world” setting [24]. Individuals who infrequently seek care or are nonadherent with medications are a specific target of the intervention.

Before the initiation of the trial, the study team developed a hypertension registry using information extracted from FNCH’s electronic medical record, eClinicalWorks (Westborough, MA). FNCH began using eClinicalWorks in 2012. The registry was built using SAS version 9.4 (SAS Institute Inc, Cary, NC). This registry had several purposes, including characterization of the FNCH population, assessment of baseline blood pressure control, and recruitment for the randomized trial. Hypertension was identified using diagnosis codes (ICD-9 401-405; ICD-10 I10-I13, I15), orders for antihypertensive medication, and elevated blood pressures (≥140/90). We considered individuals to have hypertension if any 1 of the following criteria was met: (1) having 2 visits with a hypertension diagnosis on different days, (2) having 1 visit with a hypertension diagnosis and 1 medication order, (3) having 1 visit with a hypertension diagnosis and 1 elevated blood pressure, or (4) having 2 consecutive elevated blood pressures on different days [25]. For study recruitment, we additionally required (1) at least 2 prior visits to FNCH with blood pressure measurements, at least 1 of which took place in the 24 months before recruitment, and (2) aged 21 to 79 years at the time of recruitment. Individuals aged 80 years and above were excluded because of the need to individualize treatment targets in the elderly [6]. Clients were eligible for the study even if their hypertension was under control, with the rationale that even those with controlled hypertension at a single visit may not remain in control over time. We included patients receiving care at FNCH regardless of their racial or ethnic self-identification. Individuals who resided outside Albuquerque (such as rural Indian reservations) were eligible if they identified FNCH as their primary source of care.

Exclusion criteria include the following: another preferred site of primary care, significant impairment of vision and hearing, life-limiting illnesses such as advanced cancer, renal dialysis, receipt of home health care with blood pressure monitoring and/or assistance with administration of medications, hospice services or residence in a nursing home, dementia, pregnancy at the time of recruitment, current homelessness, no landline or cellular phone access, or inability to understand English or Spanish.

Potentially eligible participants were contacted by letter and then by phone. Those who expressed interest completed an in-person baseline visit that included the informed consent process, a baseline survey, and blood pressure measurement using standardized procedures. They were then randomized to either the IVR-T intervention or usual care.

### Randomization

Individuals were randomized 1:1 to IVR-T intervention or usual care using block randomization with blocks of 4. A randomization table was generated by the study statistician (SX) using SAS software. The study coordinators were blinded to the randomization sequence.

### Intervention

Since 2005, the Institute for Health Research at Kaiser Permanente Colorado has developed and managed an IVR-T system that initiates telephone calls to landlines or cellular phones and text messages to cellular phones. The system uses a commercial database to distinguish between cellular phones and landlines and to determine whether a cellular phone is text-enabled [11]. For this intervention, information from the hypertension registry and Research Electronic Data Capture (REDCap) were used to send IVR-T messages to individuals randomized to the IVR-T intervention arm. Individuals in the intervention arm could opt to receive either automated text messages or automated phone calls in either English or Spanish. The messages include reminders of upcoming appointments at FNCH; requests to reschedule recently missed appointments; monthly reminders to refill medications; and weekly motivational messages to encourage self-care, appointment keeping, and medication taking for hypertension (Table 1; Multimedia Appendix 1). The motivational messages for text or telephone were modified from existing messages in the literature [26-29] and tailored with the help of an American Indian psychologist and the FNCH Advisory Council to be culturally appropriate. Some of the messages were recorded by the participants’ primary care clinician at FNCH. Individuals in the IVR-T arm could opt to nominate a care partner to also receive notices of upcoming and missed appointments. Individuals in the IVR-T arm were also offered a home blood pressure monitor. Blood pressure logs were not received or reviewed by the study team. The intervention is unblinded, although participants’ FNCH clinicians were not informed by the study staff of any patient’s group assignment. The protocol for IVR-T messages is shown in Table 1.

**Table 1:** Components of the interactive voice response and text message intervention.

Component	Description
Reminders for upcoming visits	Three days before and 1 day before each upcoming visit
“Recalls” after missed clinic visits	Recall 1 to 6 days after a missed visit
Reminders to refill medications	Monthly reminders to refill hypertension medications
Motivational adherence messages	Weekly, from the list in Multimedia Appendix 1
Inbound calling options	Direct dial options available to participants to reach scheduling staff, a member of the clinical team, or pharmacy to address urgent concerns during business hours

### Study Visits

Participants complete in-person baseline, 6-month, and 12-month visits. At each visit, blood pressure measurements are collected and participants complete a survey. Blood pressure is measured in a standardized fashion during the research visits, using an Omron HEM 907XL IntelliSense Professional Digital Blood Pressure Monitor (an automated sphygmomanometer) [30]. After a 5 min waiting period, blood pressures are taken 3 times, 30 seconds apart. The average of the second and third blood pressures is used. The baseline survey can be self-administered or participants can request assistance from study coordinators. The baseline survey includes questions about general health, blood pressure treatment, adherence to blood pressure medications [31], comorbidities, perceived health competence (adapted from Smith et al [32]), depression (Patient Health Questionnaire-2) [33], discrimination (adapted from several sources [34-36]), health literacy and numeracy (adapted from Brega et al [37]), exercise and diet [38], demographics, and socioeconomic status and social needs (see Multimedia Appendix 2). The 6-month and 12- month visit surveys contain a subset of the questions from the baseline survey, with additional questions about attitudes toward the intervention. Survey answers and other study data are managed using REDCap software. Participants receive US $20 gift cards for each visit.

### Baseline Variables

In addition to information from the baseline survey, we also collected demographic characteristics, hypertension characteristics, comorbidities, and geographic variables using information from the FNCH electronic health record (EHR). Race and ethnicity were categorized using the following hierarchical categories: AI/AN, Hispanic, non-Hispanic white, non-Hispanic African American, non-Hispanic other, and missing. Individuals with missing ethnicity information were assumed to be non-Hispanic.

Comorbidities such as diabetes, cardiovascular disease, and depression were defined as the presence of at least one ICD-9 or ICD-10 diagnosis code within the previous 2 years (see Multimedia Appendix 3). Chronic kidney disease was defined as being present if the most recent estimated glomerular filtration rate in the previous 2 years was less than 60 mL/min/1.73m^2^ using the CKD-EPI estimating equation [39].

Ever homeless was defined using EHR visit types specific to homeless services and include information on visits since the inception of the FNCH EHR in 2012. The multiple insurance payers for FNCH clients were categorized into 5 groups (commercial, IHS, Medicaid, Medicare, and self-pay). We then defined the dominant payer by determining which insurance category predominated over the previous 2 years. When 2 encounters had different insurance categories, we assumed that the insurance change occurred at the midpoint between the 2 encounters. We geocoded addresses and created maps to determine those in and out of the Albuquerque city limits and Bernalillo County limits using ArcMap 10.3.1. (Esri, Redlands, CA) on a secure system. We also mapped the addresses onto census tracks and reported median household incomes and educational levels, using census track information from the 2010 US census. We assessed diet quality using the diet metric from the American Heart Association Simple 7 score and physical activity using American Heart Association recommendations [38].

### Endpoints

The primary outcome of the trial is the change in systolic blood pressure between baseline and 12-month research visits, comparing participants receiving the IVR-T intervention with those in usual care. Secondary outcomes are change in diastolic blood pressure between baseline and 12 months, self-reported adherence, and the proportion of missed clinic appointments.

The primary medication adherence measure is the self-reported Voils instruments administered at baseline and during both follow-up surveys [31,40-42]. The measure has 3 questions about the extent of nonadherence over the previous 7 days; the 5 response options range from missing medication “none of the time” to missing medication “all of the time.” If 1 or more items suggest nonadherence, 21 questions probe reasons for nonadherence; the 5 response options for each question range from being “not at all” a reason for nonadherence to being “very much” a reason. In prior studies, Cronbach alpha for the 3 “extent” items was .84, and the scale correlated significantly with systolic blood pressure and diastolic blood pressure [40]. Although it would have been ideal to corroborate this measure with pharmacy refill adherence or pill counts, FNCH clients use multiple pharmacies that preclude comprehensive assessment of refill adherence [43], and performing regular pill counts was not feasible.

The measure of visit adherence is the proportion of scheduled appointments that are kept, missed, or canceled. This measure is assessed using the FNCH EHR and calculated as a proportion of all visits scheduled during the period from enrollment to the end of follow-up. This is a standard measure in interventional trials to improve appointment keeping [11]. All 3 visit outcomes (kept, missed, and canceled) are important from an operational perspective, as timely visit cancellations allow FNCH to schedule other clients.

### Data Management

Information from the hypertension registry and the randomization table was loaded into REDCap electronic data capture tools hosted at Kaiser Permanente Colorado. REDCap was then used for study data collection, data management, and completion of the study visit questionnaires. REDCap is a secure, Web-based app designed to support data capture for research studies, providing (1) an intuitive interface for validated data entry, (2) audit trails for tracking data manipulation and export procedures, (3) automated export procedures for seamless data downloads to common statistical packages, and (4) procedures for importing data from external sources [44].

### Analysis Plan

The primary analytic strategy will be intention to treat. As secondary analyses, we will also perform per protocol and as-treated analyses. Linear mixed models will be used to analyze repeated measures of systolic blood pressure (the primary outcome variable) and diastolic blood pressure (a secondary outcome variable) as continuous variables [45]. For each group, the changes from baseline at 6 and 12 months after randomization can be obtained after the linear mixed model is fit and all coefficients are estimated. Then, we will contrast the changes from baseline between the 2 groups to assess the influence of IVR-T intervention on systolic blood pressure and diastolic blood pressure. We also plan to analyze the secondary outcomes missed appointment and medication adherence status using nonlinear mixed models.

We anticipate that some outcomes will be missing because of missed appointments or attrition. By default, the linear mixed models accommodate missing data by assuming that data are missing at random (MAR). We will explore different ways to examine the assumption of MAR [46]. When the data are missing not at random, outcome measures and missingness will be jointly modeled with either random effects selection models or pattern mixture models, whichever is appropriate [47,48]. Multiple imputation methods will also be used to explore the sensitivity of the results to missingness [49].

In our analyses of the baseline data, we compared individuals in the recruitment pool who did and did not enroll in the trial using SAS version 9.4. Statistical significance was computed using Fisher exact test for categorical variables and Mann-Whitney for discrete ordinal variables and continuous variables. A *t* test was used to compare with Mann-Whitney *P* values as a check, and the results were similar except for distance to clinic. Due to outliers for distance to clinic, we report the medians. To compare agreement between the EHR and the study survey for specific comorbidities, we used the prevalence and bias adjusted kappa [50].

### Power

Our initial target sample size for this study was 512, based on the following assumptions: (1) 1800 individuals in the FNCH hypertension registry, (2) 36% of eligible FNCH clients would agree to enter the study, and (3) 80% of those would complete the trial. This would have yielded 80% statistical power to detect a 5 mmHg reduction in systolic blood pressure with a Cronbach alpha of .05 and estimated SD in systolic blood pressure of 18 mmHg [51].

### Ethical Oversight

This study was approved by the Kaiser Permanente Colorado Institutional Review Board (KPCO IRB). The Colorado Multiple Institutional Review Board, which governs research activities at the University of Colorado Anschutz Medical Campus, ceded to the KPCO IRB. The National Indian Health Service Institutional Review Board determined that it did not have oversight of the study and deferred to local authorities. The study was registered at clinicaltrials.gov (#NCT03135405) on April 27, 2017.

## Results

### Participant Recruitment

Recruitment for the study took place from April 12, 2017, to January 31, 2018. Out of a recruitment list of 1497, a total of 295 eligible individuals completed the informed consent process and were randomized. An additional 6 individuals were randomized but were then found to be ineligible, as they had not had a qualifying visit at FNCH in the previous 2 years. These individuals will be excluded from all analyses. Individuals were not enrolled because of the following reasons: could not be contacted (n=508 or 35.0%), declined to participate (n=496 or 33.1%), were ineligible (n=106 or 7.1%), or never completed a baseline visit (n=92 or 6.1%; see Multimedia Appendix 4).

### Comparison of Eligible and Enrolled Individuals

A total of 1497 potentially eligible individuals were identified through the hypertension registry. Overall, the differences were small between those enrolled in the RCT and FNCH patients with hypertension who were ineligible, could not be reached, or chose not to enroll (Table 2). There was a slightly greater proportion of women in the enrolled group. The enrolled group also had a higher proportion of Hispanic individuals. Enrolled individuals had a slightly lower blood pressure than those not enrolled, but the mean difference was small (2.6/1.7 mmHg). The largest difference between the groups is that the enrolled group was more likely to self-pay for their care (26.1% vs 10.0%) and had a more recent primary care visit at FNCH (164 days vs 231 days). Although we excluded individuals who were homeless at the time of enrollment, 14.2% of individuals had received homeless services from FNCH since 2012.

### Baseline Characteristics of the Study Cohort

Baseline characteristics of the study cohort are reported in Tables 2-4. Table 2 is derived from the FNCH EHR, whereas Tables 3 and 4 are self-reported information from the baseline survey. The number of missing responses per survey question was 8 or less, except for income (number missing=30), the perceived health competence scale (number missing=15), and specific items of the basic material needs assessment (see footnote to Table 4).

**Table 2:** Individuals in the First Nations Community Health Source hypertension registry, by enrollment status (N=1497).

Characteristics			Enrolled in the RCT^a^ (study cohort; n=295)	Not enrolled in the RCT (n=1202)	Total (recruitment pool; n=1497)	*P* value
**Demographics**						
	**Age (years), n (%)**					.71
		18-44	71 (24.1)	278 (23.1)	349 (23.3)	—
		45-64	169 (57.3)	674 (56.1)	843 (56.3)	—
		≥65	55 (18.6)	250 (20.8)	305 (20.4)	—
	Age (years), mean (SD)		53.4 (11.3)	53.6 (12.5)	53.6 (12.2)	.52
	Female, n (%)		176 (59.7)	637 (53.0)	813 (54.3)	.04
	**Race, n (%)**					<.001
		AI/AN^b^	65 (22.0)	358 (29.8)	423 (28.3)	—
		Hispanic	155 (52.5)	442 (36.8)	597 (39.9)	—
		Non-Hispanic white	47 (15.9)	248 (20.6)	295 (19.7)	—
		Non-Hispanic African American	18 (6.1)	83 (6.9)	101 (6.8)	—
		Other	7 (2.4)	53 (4.4)	60 (4.0)	—
		Missing	3 (1.0)	18 (1.5)	21 (1.4)	—
	BMI^c^, mean (SD); number of people with missing values		32.6 (7.3); 1	32.2 (7.9); 23	32.2 (7.8); 24	.21
	Time between last clinic visit and randomization date in days, mean (SD)		164.3 (174.8)	230.9 (217.9)	217.8 (211.8)	<.001
	**Insurance, n (%)**					<.001
		Commercial	111 (37.6)	544 (45.3)	655 (43.8)	—
		IHS^d^	9 (3.1)	82 (6.8)	91 (6.1)	—
		Medicaid	46 (15.6)	207 (17.2)	253 (16.9)	—
		Medicare	46 (15.6)	227 (18.9)	273 (18.2)	—
		Self-pay	77 (26.1)	120 (10.0)	197 (13.2)	—
		Unknown	6 (2.0)	22 (1.8)	28 (1.9)	—
	Ever homeless, n (%)		47 (15.9)	165 (13.7)	212 (14.2)	.35
**Hypertension characteristics**						—
	Hypertension diagnosis in EHR^e^, n (%)		271 (91.9)	1074 (89.4)	1345 (89.8)	.24
	Hypertension medication order in EHR, n (%)		260 (88.1)	1042 (86.7)	1302 (87.0)	.56
	Most recent SBP^f,g^, mmHg, mean (SD)		129.3 (13.9)	131.9 (17.3)	131.4 (16.7)	.04
		Most recent DBP^g,h^, mmHg, mean (SD)	77.0 (9.8)	78.7 (10.6)	78.4 (10.5)	.01
		SBP≥140 or DBP≥90, n (%)	72 (24.4)	373 (31.0)	445 (29.7)	.03
**Comorbidities, n (%)**						—
	Diabetes		97 (32.9)	385 (32.0)	482 (32.2)	.78
	Cardiovascular disease		14 (4.7)	39 (3.2)	53 (3.5)	.22
	Depression		77 (26.1)	256 (21.3)	333 (22.2)	.09
	Chronic kidney disease		31 (12.7)	117 (13.1)	148 (13.0)	.91
**Geographic variables^i^**						—
	Census track median household income, mean (SD)		US $35,900 (14,199.9)	US $35,735 (16,186.5)	US $35,768 (15,808.6)	.27
	Percent of census track with less than high school education, mean (SD)		17.6 (0.11)	17.4 (0.11)	17.4 (0.11)	.59
	Distance from home address to clinic (miles), median (IQR^j^)		3.89 (1.62-6.76)	3.91 (1.64-7.56)	3.91 (1.63-7.47)	.65
	Live in the City of Albuquerque, n (%)		269 (91.2)	1,070 (89.0)	1,339 (89.4)	.29
	Live in Bernalillo County, n (%)		283 (95.9)	1,122 (93.3)	1,405 (93.9)	.11

^a^RCT: randomized controlled trial.

^b^AI/AN: American Indian or Alaska Native.

^c^BMI: body mass index.

^d^IHS: Indian Health Service.

^e^EHR: electronic health record.

^f^SBP: systolic blood pressure.

^g^Primary care visits only.

^h^DBP: diastolic blood pressure.

^i^12 individuals had addresses that could not be geocoded, 1 in the RCT group and 11 in the not enrolled group.

^j^IQR: interquartile range.

**Table 3:** Baseline demographic characteristics of the study cohort (N=295).

Characteristics		Statistics
**Gender, n (%)**		
	Male	117 (39.7)
	Female	175 (59.3)
	Other	3 (1.0)
Primary language: Spanish, n (%)		116 (39.3)
**Race, n (%)**		
	American Indians and Alaska Natives	74 (25.1)
	Hispanic	153 (51.9)
	Non-Hispanic white	43 (14.2)
	Non-Hispanic African American	14 (4.8)
	Other	11 (3.7)
**Marital status, n (%)^a^**		
	Married	95 (32.3)
	Marriage-like relationship	37 (12.6)
	Separated or divorced	83 (28.2)
	Widowed	20 (6.8)
	Never married	59 (20.1)
**Education, n (%)^a^**		
	8th grade or less	79 (26.9)
	Some high school	32 (10.9)
	High school grade or General Equivalent Development Test	61 (20.8)
	Some college	79 (26.9)
	4-year college degree	25 (8.5)
	More than 4-year college degree	18 (6.1)
**Employment, n (%)**		
	Employed for wages	106 (35.9)
	Self-employed	20 (6.8)
	Out of work for 1 year or more	19 (6.4)
	Out of work for less than 1 year	12 (4.1)
	Homemaker	32 (10.9)
	Student	4 (1.4)
	Retired	50 (17.0)
	Unable to work	52 (17.6)
**Household income, n (%)^a^**		
	Nothing	27 (10.2)
	Less than US $10,000	75 (28.3)
	US $10,000 to US $14,999	50 (18.9)
	US $15,000 to US $19,999	26 (9.8)
	US $20,000 to US $24,999	35 (13.2)
	US $25,000 to US $34,999	22 (8.3)
	US $35,000 to US $49,999	17 (6.4)
	US $50,000 to US $74,999	10 (3.8)
	More than US $75,000	3 (1.1)
Household income, mean (SD)		US $17,170 (17,058)

^a^Numbers do not sum to 295 due to missing values. The number of missing values are as follows: marital status=1, education=1, household income=30.

Self-reported race/ethnicity differed somewhat from race/ethnicity data from the EHR, although the overall agreement was 90.2% (266/295). The study cohort was 25.1% (74/295) AI/AN and 51.9% (153/295) Hispanic. Among the AI/AN participants, 8 (10.8%) also identified as Hispanic. Almost all participants reported having hypertension (94.5%), whereas 79.8% reported taking blood pressure medication. Only 17.4% had a home blood pressure monitor. Nearly all individuals in the intervention group (144/148, 97%) opted to receive a blood pressure monitor as part of the study intervention. Over one-third (39.0%) had a baseline blood pressure greater than or equal to 140/90 mmHg. Our self-reported medication adherence rate was 36.4%. Concordance between the medical record and the survey was relatively good for most chronic health conditions, with prevalence and bias adjusted kappa for diabetes, CVD, chronic kidney disease, and depression of 0.81, 0.71, 0.73, and 0.55, respectively.

Over three-quarters of the sample reported a poor diet. Our study cohort reported poor general health, with only 46% reporting good or better health. Self-reported socioeconomic status was low. Over one-third of participants had less than a high school education, and over one-quarter of individuals were unemployed or unable to work. Over one-third of participants reported annual household incomes of less than US $10,000, with a mean annual household income of US $17,170 and median household income of US $12,500. Basic resource needs were very common. High percentages of participants reported they did not always have enough money to buy food, health care, or utilities.

Individuals who did not answer or who chose the “does not apply” option were included in the denominator as not having that particular social need; this number varied widely between the different options (clothes=1, place to live=2, utilities=17, childcare=227, debts=87, transportation=4, and Supplemental Nutrition Assistance Program=1).

Our recruitment pool from the FNCH hypertension registry was 17% smaller than originally estimated (1497 instead of 1800). We successfully recruited 295 individuals or 20%. Multimedia Appendix 5 shows the number of individuals recruited as a function of time; we were able to recruit 8 participants per week through most of the study period. By the end of the recruitment period, we had exhausted the recruitment pool, with very few individuals being added to the recruitment registry each week. With our final sample of 295 participants, we have 80% statistical power to detect a 6.5 mm Hg reduction in systolic blood pressure with a Cronbach alpha of .05 and an estimated SD in systolic BP of 18 mmHg.

**Table 4:** Baseline health status and psychosocial characteristics of the study cohort (N=295).

Characteristics			Statistics
**Hypertension characteristics**			
	Report having hypertension, n (%); number of people with missing values		277 (94.5); 2
	Taking blood pressure medications, n (%); number of people with missing values		233 (79.8); 3
	Having a home blood pressure monitor, n (%); number of people with missing values		51 (17.4); 2
	Systolic blood pressure at enrollment visit (mmHg), mean (SD)		133.6 (19.5)
	Diastolic blood pressure at enrollment visit (mmHg), mean (SD)		81.5 (12.6)
	Elevated blood pressure (SBP≥140 or DBP≥90 mmHg), n (%)		115 (39.0)
	Adherent to blood pressure medications (Voils adherence scale), n (%); number of people with missing values		82 (36.4); 8
**Self-reported comorbid conditions**			
	Prediabetes, n (%)		57 (19.3)
	Diabetes, n (%)		87 (29.4)
	Heart disease, n (%)		51 (17.3)
	Kidney disease, n (%)		25 (8.5)
	Depression, n (%)		109 (36.9)
	Positive screening Patient Health Questionnaire-2, n (%); number of people with missing values		69 (23.8); 5
	Arthritis, n (%)		72 (24.4)
	Back pain, n (%)		123 (41.7)
**Health status and self-care behaviors**			
	**General health, n (%)^a^**		
		Excellent	4 (1.4)
		Very good	29 (9.9)
		Good	101 (34.6)
		Fair	130 (44.5)
		Poor	28 (9.6)
	**AHA^b^ Healthy 7–diet component, n (%)^a^**		
		Poor	221 (75.2)
		Intermediate	68 (23.1)
		Ideal	5 (1.7)
	**Physical activity, n (%)^a^**		
		None	28 (9.5)
		Intermediate	177 (60.2)
		Ideal	89 (30.3)
	Health literacy (possible range 1-5), mean (SD)		3.85 (0.95)
	Health numeracy (possible range 0-1), mean (SD); number of people with missing values		0.61 (0.28); 8
	Perceived health competence scale (possible range 1-5), mean (SD); number of people with missing values		3.31 (0.60); 15
**Discrimination due to race or ethnicity**			
	**Experience discrimination, n (%)**		
		Never	116 (39.3)
		Once or twice	50 (17.0)
		A few times	86 (29.2)
		Many times	37 (12.5)
		All the time	6 (2.0)
	**Experience discrimination in health care setting, n (%)**		
		Never	220 (74.6)
		Once or twice	45 (15.3)
		A few times	21 (7.1)
		Many times	9 (3.1)
		All the time	0 (0)
	**Family members experience discrimination, n (%)^a^**		
		Never	137 (47.1)
		Once or twice	43 (14.8)
		A few times	61 (21.0)
		Many times	44 (15.1)
		All the time	6 (2.1)
	**Family members experience discrimination in health care setting, n (%)^a^**		
		Never	219 (75.0)
		Once or twice	34 (11.6)
		A few times	31 (10.6)
		Many times	7 (2.4)
		All the time	1 (0.3)
**Substance use**			
	**Alcohol, n (%)**		
		Never	149 (50.5)
		Monthly or less	66 (22.4)
		2 to 4 times per month	45 (15.3)
		2 to 3 times per week	20 (6.8)
		4 or more times per week	15 (5.1)
	**Tobacco, n (%)^a^**		
		Yes	75 (25.5)
		Yes, only ceremonial purposes	9 (3.1)
		No, I quit within the last year	11 (3.7)
		No, I quit over a year ago	58 (19.7)
		Never	141 (48.0)
	**Illegal drugs, n (%)**		
		Never	266 (90.2)
		Sometimes	23 (7.8)
		Often	3 (1.0)
		Very often	3 (1.0)
**Basic resource needs, n (%)^c^**			
	Food		99 (33.6)
	Health care		123 (41.7)
	Clothes		74 (25.1)
	Place to live		50 (16.9)
	Utilities		158 (53.6)
	Childcare		37 (12.5)
	Debts		144 (48.8)
	Transportation		74 (25.1)
	Enrolled in Supplemental Nutrition Assistance Program		142 (48.1)

^a^Numbers do not sum to 295 due to missing values. The number of missing values are as follows: general health=3, AHA healthy 7-diet component=1, physical activity=1, family members experience discrimination=4, family members experience discrimination in health care setting=3, and tobacco=1.

^b^AHA: American Heart Association.

^c^Individuals who did not answer or who chose the “does not apply” option were included in the denominator as not having that particular social need. This number was: clothes=1, place to live=2, utilities=17, childcare=227, debts=87, transportation=4, and Supplemental Nutrition Assistance Program=1.

## Discussion

Improving the health of underserved populations requires effective, low-resource interventions. The Controlling Blood Pressure Trial will evaluate the effectiveness of an IVR-T intervention to improve blood pressure, medication adherence, and visit keeping among individuals with hypertension who receive care at a UIHO. The study has enrolled 295 individuals with hypertension. Important design features of this trial include (1) registry-based recruitment, which allows the assessment of generalizability to the source population, and (2) a pragmatic design that minimizes exclusions.

A recent review of the representativeness of major cardiology randomized clinical trials found that “real-world” cardiology patients tend to have higher risk characteristics, to be older, to be more likely to be female, to have clinical impairments and comorbidity disease, and to be treated less frequently with guidelines-recommended therapy, compared with individuals enrolled in cardiology RCTs. In many studies, over 50% of “real-world” patients would be ineligible for trials [52]. However, in many cases, the actual source population for a given study is unknown, and assumptions must be made in extrapolating the characteristics of study participants to the source population [53,54]. Through registry-based recruitment, we were able to precisely define the source population for our trial. We efficiently recruited individuals through the use of an EHR-based registry. Overall, the study population is representative of the eligible clinic population in its level of blood pressure control and most sociodemographic characteristics, although it does represent a group that is more likely to self-pay for their care and has been seen more recently at FNCH.

The study is designed to be pragmatic [55], with inclusive eligibility criteria. We designed the intervention itself to be flexible, allowing participants to choose the mode of delivery (text messages or phone calls), language of delivery (English or Spanish), whether to include a care partner, and whether to receive a home blood pressure monitor. The intervention included both mandatory comments (educational messages and visit reminders), and optional components (care partner and home blood pressure monitors), which replicates clinical practice. Due to the pragmatic nature of the trial, we are relying on self-assessment of medication adherence for secondary outcomes. Like most clinicians, we were unable to conduct pill counts or calculate medication refill adherence, as participants receive medications from multiple pharmacies.

We did observe some discrepancies in baseline data between the 2 data sources (self-report vs EHR). For example, the race data showed 90.2% agreement between the data sources. For the main trial analysis, self-reported race will be used as the gold standard. The proportions of those reporting having hypertension and taking blood pressure medications (94.5% and 79.8%, respectively) were similar to those found from the EHR data (91.9% and 88.1%, respectively). Differences may be due to a number of factors, including incomplete medical coding, individuals not taking medications that have been prescribed, blood pressure medications that have other indications, and individuals not understanding the indications for all their medications.

The trial is being conducted in a clinic with a population that is largely underserved, without substantial additional resources from the clinic itself. On the baseline survey, we confirmed that the study population is a group with high medication nonadherence, poor self-reported health, low socioeconomic status, and high social needs. Other characteristics such as health literacy, health numeracy, and diet are similar to populations in other safety-net health systems. To put our self-reported medication adherence rate of 36.4% in perspective, Weidenbacher et al found an adherence rate of 63% among US veterans taking antihypertensive medications, using the same adherence scale [41]. Our percent reporting good or better health (46%) can be compared with 2016 Behavioral Risk Factor Surveillance System rates of 78% in New Mexico and 79% in the Albuquerque Metropolitan Area [56].

Our finding that over three-quarters of the sample reported a poor diet score is similar to other studies. National Health and Nutrition Examination Survey (NHANES) 2005-2006 found that 76% of adults reported a poor diet, 24% an intermediate diet, and <0.5% an ideal diet [38]. In the Strong Heart Family study, among a sample of 1639 American Indians without diabetes at the baseline exam, 8.1% reported an intermediate diet score and 91.9% a poor diet score [56]. The health literacy and numeracy scores we found are similar to those from a survey of 3033 American Indian and Alaska Native adults with diabetes who were enrolled in the Special Diabetes Program for Indians Healthy Heart Project, with our population having a slightly higher print literacy and slightly lower health numeracy [37]. Mean perceived health competence was similar to a study based in a Kentucky practice-based research network on individuals with diabetes and suboptimal blood pressure control (3.3 vs 3.2) [57].

We found a baseline level of blood pressure control (blood pressure <140/90) of 61.0%, which compares favorably with the national NHANES 2011 to 2014 estimates of 54.4% [58]. Including individuals with blood pressure at goal at baseline will limit our power to detect a difference between the intervention and usual care groups but reflects the pragmatic design of the trial. Although we focused on designing an intervention that was feasible in the UIHO setting, we enrolled individuals regardless of race and ethnicity. We enrolled a slightly smaller proportion of AI/ANs than in the recruitment pool (22.0% vs 28.3%). This will also limit our ability to detect interactions by race, but again reflects the pragmatic design of the trial and recommendations from the Advisory Council.

Hypertension is the most common chronic disease in the United States, and treatment of hypertension is crucial to CVD prevention. Although the Controlling Blood Pressure Trial is set in a UIHO, it could potentially be adapted to other under-resourced clinical environments. It, therefore, has implications for other populations with low socioeconomic status and high social needs. Results from the trial should be available in early 2019.

## Appendix

Multimedia Appendix 1 Messaging used for the IVR-T intervention.

Multimedia Appendix 2 Baseline survey.

Multimedia Appendix 3 Diagnosis codes for hypertension, diabetes, cardiovascular disease, and depression.

Multimedia Appendix 4 Recruitment Diagram.

Multimedia Appendix 5 Recruitment plot.

## Abbreviations

AAAHC: Accreditation Association for Ambulatory Health Care
AI/ANs: American Indians and Alaska Natives
CAIANH: Centers for American Indian and Alaska Native Health
CVD: cardiovascular disease
EHR: electronic health record
FNCH: First Nations Community HealthSource
IHS: Indian Health Service
IVR-T: interactive voice response and text message
KPCO IRB: Kaiser Permanente Colorado Institutional Review Board
MAR: missing at random
NHANES: National Health and Nutrition Examination Survey
NIDDK: National Institute for Diabetes and Digestive and Kidney Diseases
RCTs: randomized controlled trials
REDCap: research electronic data capture
UIHO: Urban Indian Health Organization
