# Supplementary material for: An Interactive Voice Response and Text Message Intervention to Improve Blood Pressure Control Among Individuals With Hypertension Receiving Care at an Urban Indian Health Organization: Protocol and Baseline Characteristics of a Pragmatic Randomized Controlled Trial
Source: JMIR Res Protoc. 2019 Apr 2;8(4):e11794. doi: 10.2196/11794 (PMC6465973; doi:10.2196/11794)
Supplement: Multimedia Appendix 1 [file resprot_v8i4e11794_app1.pdf]

Multimedia Appendix 1. Messaging used for the IVR-T intervention.

English - Telephone	Spanish - Telephone	English - SMS	Spanish - SMS
**General motivational messages**			
Remember that high blood pressure can increase your chance of having a heart attack or stroke. Ask your provider what your goal blood pressure is.	Recuerde que la presión alta puede aumentar la probabilidad de desarrollar un ataque al corazón o un derrame cerebral. Pregúntele a su doctor o doctora cuál debe ser su meta al medirse la presión.	High blood pressure can increase your chance of having a heart attack or stroke. Ask your provider what your goal blood pressure is.	Recuerde que la presión alta puede aumentar la probabilidad de desarrollar un ataque al corazón o un derrame cerebral. Pregúntele a su doctor/a cuál debe ser su meta al medirse la presión.
Contact your provider at #FacTelNo# if you have chest pain, shortness of breath, or swollen legs. [1]	Comuníquese con su doctor/a al #FacTelNo# si tiene dolor de pecho, falta de aire o las piernas hinchadas.	Contact your provider at #FacilityTelNo# if you have chest pain, shortness of breath, or swollen legs.	Comuníquese con su doctor/a al #FacTelNo# si tiene dolor de pecho, falta de aire o las piernas hinchadas.
You’re doing very well. Please keep on with your pills, come to your clinic appointments, exercise and eat healthy food. [1,2]	Está haciendo las cosas muy bien. Siga tomando sus pastillas, acuda a sus citas en la clínica, haga ejercicio y coma alimentos saludables.	You’re doing very well. Please keep on with your pills, come to your clinic appointments, exercise and eat healthy food.	Está haciendo todo muy bien. Siga tomando sus pastillas, acuda a sus citas en la clínica, haga ejercicio y coma alimentos saludables.
**Adherence**			
A pill box can help you to remember when to take your high blood pressure pills. You can get one at the pharmacy.	Una cajita para organizar sus pastillas puede ayudarle a recordar cuándo debe tomar sus medicinas para la presión. Puede conseguir una en la farmacia.	A pill box can help you to remember when to take your high blood pressure pills. You can get one at the pharmacy.	Una cajita para organizar sus pastillas puede ayudarle a recordar cuándo debe tomar sus medicinas para la presión alta. Puede conseguir una en la farmacia.
Make taking your pills part of your daily routine, like when you first wake up. Doing this can help you remember to take them regularly. [2]	Tomar sus pastillas debe formar parte de su rutina diaria, por ejemplo, tómelas al despertarse. Esto puede ayudarle a recordar que debe	Make taking your pills part of your daily routine, like when you first wake up. Doing this can help you remember to take them regularly.	Tomar sus pastillas debe formar parte de su rutina diaria, por ejemplo, tómelas al despertarse. Esto puede ayudarle a recordar que debe

	tomarlas regularmente.		tomarlas regularmente.
Ask someone you trust to help you remember to take your medicine as directed. [2]	Pídale a alguien de confianza que le ayude a recordar que debe tomar sus medicinas como se le indicó.	Ask someone you trust to help you remember to take your medicine as directed.	Pídale a alguien de confianza que le ayude a recordar que debe tomar sus medicinas como se le indicó.
Don't stop taking your medication because you feel better. Remember you can have high blood pressure without having symptoms. [1]	No deje de tomar sus medicamentos porque se siente mejor. Recuerde que puede tener la presión alta y no tener síntomas.	Don't stop taking your medication because you feel better. Remember you can have high blood pressure without having symptoms.	No deje de tomar sus medicamentos porque se siente mejor. Recuerde que puede tener la presión alta y no tener síntomas.
If you are afraid of being dependent on your high blood pressure pills, please talk with your provider or pharmacist about your concerns.	Si tiene miedo que las pastillas para la presión alta le causen dependencia, por favor, hable con su doctor/a o farmacéutico/a sobre sus inquietudes.	If you are afraid of being dependent on your high blood pressure pills, please talk with your provider or pharmacist about your concerns.	Si tiene miedo que las pastillas para la presión alta le causen dependencia, por favor, hable con su doctor/a o farmacéutico/a sobre sus inquietudes.
If you are experiencing a side effect from your high blood pressure pills, please talk with your provider or pharmacist. [2]	Si sus pastillas para la presión le ocasionan algún efecto secundario, por favor, hable con su doctor/a o farmacéutico/a	If you are experiencing a side effect from your high blood pressure pills, please talk with your provider or pharmacist.	Si sus pastillas para la presión le ocasionan algún efecto secundario, por favor, hable con su doctor/a o farmacéutico/a
**Stress management**			
To decrease stress: Breathe in deeply for 4 seconds and out slowly for 4 seconds.	Para disminuir el estrés: aspire el aliento profundamente durante 4 segundos y exhale lentamente durante 4 segundos.	To decrease stress: Breathe in deeply for 4 seconds and out slowly for 4 seconds.	Para disminuir el estrés: aspire el aliento profundamente durante 4 segundos y exhale lentamente durante 4 segundos.
To decrease stress: Take a mental vacation – spend 3 minutes in your favorite place.	Para disminuir el estrés: tómese vacaciones mentales, imagínese que está en su lugar favorito durante 3 minutos.	To decrease stress: Take a mental vacation – spend 3 minutes in your favorite place.	Para disminuir el estrés: tómese vacaciones mentales, imagínese que está en su lugar favorito durante 3 minutos.
To decrease stress: Imagine the last time	Para disminuir el estrés: imagine la	To decrease stress: Imagine the last time	Para disminuir el estrés: imagine la

you laughed really hard – now laugh twice as hard.	última vez que se rio a carcajadas, ahora ríase el doble de fuerte.	you laughed really hard – now laugh twice as hard.	última vez que se rio a carcajadas, ahora ríase el doble de fuerte.
To decrease stress: Listen to some of your favorite music or go for a walk.	Para disminuir el estrés: escuche su música favorita o salga a caminar.	To decrease stress: Listen to some of your favorite music or go for a walk.	Para disminuir el estrés: escuche su música favorita o salga a caminar.
To decrease stress: Put your hand over your heart and feel it slowly calm down.	Para disminuir el estrés: Coloque la mano sobre el corazón y sienta como se calma lentamente.	Put your hand over your heart and feel it slowly calm down.	Para disminuir el estrés: Coloque la mano sobre el corazón y sienta como se calma lentamente.
**Coping with change**			
Adding healthy habits takes time. Even small changes can help.	Añadir costumbres saludables toma tiempo. Hasta los cambios pequeños pueden ayudar.	Adding healthy habits takes time. Even small changes can help.	Añadir costumbres saludables toma tiempo. Hasta los cambios pequeños pueden ayudar.
Adding healthy habits takes time. Ask for help at your clinic – the team cares for you.	Añadir costumbres saludables toma tiempo. Solicite ayuda en su clínica, el equipo se preocupa por usted.	Adding health habits takes time. Ask for help at your clinic – the team cares for you.	Añadir costumbres saludables toma tiempo. Solicite ayuda en su clínica, el equipo se preocupa por usted.
**Inspiration**			
A healthy family starts with a healthy you. Keep up the good work.	La buena salud de su familia comienza cuando usted disfruta de buena salud. Siga así.	A healthy family starts with a healthy you. Keep up the good work.	La buena salud de su familia comienza cuando usted disfruta de buena salud. Siga así.
A healthy community starts with a healthy you. Start by making small changes to improve your health.	Una comunidad saludable comienza con usted. Empiece a hacer pequeños cambios para mejorar su salud.	A healthy community starts with a healthy you. Start by making small changes to improve your health.	Una comunidad saludable comienza con usted. Empiece a hacer pequeños cambios para mejorar su salud.
Good health gives us more energy to play with our kids and grandkids. Keep up the good work.	La buena salud nos da más energía para jugar con nuestros hijos y nietos. Siga así.	Good health gives us more energy to play with our kids and grandkids. Keep up the good work.	La buena salud nos da más energía para jugar con nuestros hijos y nietos. Siga así.
Remember that you have people to turn to when times get tough. Call on a friend, family member, or your health care team.	Recuerde que cuenta con personas a las que puede recurrir en situaciones difíciles. Llame a un amigo, a un pariente o a su	You have people to turn to when times get tough, a friend, family member, or your health care team.	Recuerde que cuenta con personas a las que puede recurrir en situaciones difíciles. Llame a un amigo, a un pariente o a su

	equipo de atención médica.		equipo de atención médica.
Our ancestors have a history of healthy eating. Try to eat more traditionally and healthy.	Nuestros antepasados se alimentaban de manera saludable. Intente comer de forma más tradicional y saludable.	Our ancestors have a history of healthy eating. Try to eat more traditionally and healthy.	Nuestros antepasados se alimentaban de manera saludable. Intente comer de forma más tradicional y saludable.
Our ancestors have a history of healthy eating. Eat only what sustains you.	Nuestros antepasados se alimentaban de manera saludable. Acostúmbrese a comer solamente lo que le sustenta.	Our ancestors have a history of healthy eating. Eat only what sustains you.	Nuestros antepasados se alimentaban de manera saludable. Acostúmbrese a comer solamente lo que le sustenta.
**Diet – salt intake**			
Foods high in salt can increase blood pressure. Taste food before you add salt. [1]	Los alimentos con alto contenido de sal pueden subir la presión. Pruebe los alimentos antes de agregarles sal.	Foods high in salt can increase blood pressure. Taste food before you add salt.	Los alimentos con alto contenido de sal pueden subir la presión. Pruebe los alimentos antes de agregarles sal.
Reduce how much salt you cook with little by little. That way you and your family will get used to it. [3]	Reduzca poco a poco la sal que le añade a la comida cuando cocina; de esta manera, usted y su familia se acostumbrarán.	Reduce how much salt you cook with little by little. That way you and your family will get used to it.	Reduzca poco a poco la sal que le añade a la comida cuando cocina; de esta manera, usted y su familia se acostumbrarán.
To help reduce salt intake, use spices instead of salt in cooking and at the table. Flavor foods with herbs, citrus, vinegar, or salt-free blends. [4]	Para reducir la sal en la comida, reemplace la sal por especias, en la cocina y en la mesa. Sazone las comidas con hierbas, jugo de limón, vinagre o especias sin sal.	To help reduce salt intake, use spicies instead of salt in cooking and at the table. Flavor foods with herbs, citrus, vinegar, or salt-free blends.	Para reducir la sal en la comida, reemplace la sal por especias, en la cocina y en la mesa. Sazone las comidas con hierbas, jugo de limón, vinagre o especias sin sal.
To help reduce salt intake, try to eat less canned vegetables. Eat frozen or fresh vegetables instead.	Para ayudar a reducir la sal en la comida, pruebe comer menos verduras enlatadas. Seleccione verduras congeladas o frescas.	To help reduce salt intake, try to eat less canned vegetables. Eat frozen or fresh vegetables instead.	Para ayudar a reducir la sal en la comida, pruebe comer menos verduras enlatadas. Seleccione verduras congeladas o frescas.
**Diet – other measures**			

Take care of your family by setting a good example. Try to eat 5 portions of fruits and vegetables every day. Put vegetables on your rice, pasta, and stews. [3]	Cuide a su familia poniendo un buen ejemplo. Trate de comer 5 porciones de frutas y verduras todos los días. Añada verduras al arroz, la pasta y los guisos.	Take care of your family by setting a good example. Try to eat 5 portions of fruits and vegetables every day. Put vegetables on your rice, pasta, and stews.	Cuide a su familia poniendo el ejemplo. Trate de comer 5 porciones de frutas y verduras todos los días. Añada verduras al arroz, la pasta y los guisos.
Feeling like snacking? Try a raw carrot, some tomatoes or cucumbers or a fruit. These are healthier than chips and cookies. [1]	¿Se le antoja comer un bocadillo o botana? Cómase una zanahoria cruda, algunos tomates, pepinos o una fruta, que son más saludables que las papas fritas y las galletas dulces.	Feeling like snacking? Try a raw carrot, some tomatoes or cucumbers or a fruit. These are healthier than chips and cookies.	¿Se le antoja comer un bocadillo o botana? Cómase una zanahoria cruda, algunos tomates, pepinos o una fruta, que son más saludables que las papas fritas y las galletas dulces.
Fast food is often high in fat and salt. Instead of fries, try a salad. [1]	La comida rápida o comida chatarra, por lo general, tiene mucha grasa y sal. En lugar de papas fritas, coma una ensalada.	Fast food is often high in fat and salt. Instead of fries, try a salad.	La comida rápida o comida chatarra, por lo general, tiene mucha grasa y sal. En lugar de papas fritas, coma una ensalada.
To avoid gaining weight, drink water instead of pop, juices, or other sugar sweetened drinks. [3]	Para evitar subir de peso, beba agua en lugar de refrescos, jugos u otras bebidas endulzadas con azúcar.	To avoid gaining weight, drink water instead of pop, juices, or other sugar-sweetened drinks.	Para evitar subir de peso, beba agua en lugar de refrescos, jugos u otras bebidas endulzadas con azúcar.
**Physical activity and weight management**			
Did you know that blood pressure rises as your weight increases? Losing even a small amount of weight can lower blood pressure. [4]	¿Sabe usted que a medida que aumenta de peso aumenta su presión? Perder un poco de peso puede ayudar a bajar su presión.	Did you know that blood pressure rises as your weight increases? Losing even a small amount of weight can lower blood pressure.	¿Sabe usted que a medida que aumenta de peso aumenta su presión? Perder un poco de peso puede ayudar a bajar su presión.
Physical activity lowers blood pressure. Try a 30 minute walk or three 10 minute walks. Every little bit helps [4]	El ejercicio baja la presión. Pruebe caminar 30 minutos al día. Si prefiere, camine 10 minutos 3 veces al día. Todo ayuda.	Physical activity lowers blood pressure. Try a 30 minute walk or three 10 minute walks. Every little bit helps.	El ejercicio baja la presión. Pruebe caminar 30 minutos al día. Si prefiere, camine 10 minutos 3 veces al día. Todo ayuda.

Be active. Physical activity is one of the best ways to lower your stress. You can walk or exercise at home. [1]	Manténgase activo. El ejercicio es una de las mejores maneras de disminuir el estrés. Puede caminar o hacer ejercicio en casa.	Be active. Physical activity is one of the best ways to lower your stress. You can walk or exercise at home.	Manténgase activo. El ejercicio es una de las mejores maneras de disminuir el estrés. Puede caminar o hacer ejercicio en casa.
Try to do 30 minutes of physical activity every day. Start by doing something easy for you and increase the time little by little. [3]	Trate de hacer 30 minutos de ejercicio todos los días. Empiece con algo que le resulte fácil y aumente el tiempo poco a poco.	Try to do 30 minutes of physical activity every day. Start by doing something easy for you and increase the time little by little.	Trate de hacer 30 minutos de ejercicio todos los días. Empiece con algo que le resulte fácil y aumente el tiempo poco a poco.
**Blood pressure monitoring**			
Keeping track of your blood pressure at home can help you see how you are doing. Let your provider know if your blood pressure is high.	Anotar los resultados de su presión al medírsela en su casa puede ayudarle a ver si mejora o empeora. Si su presión está alta, avísele a su doctor/a.	Keeping track of your blood pressure at home can help you see how you are doing. Let your provider know if your blood pressure is high.	Anotar los resultados de su presión al medírsela en su casa puede ayudarle a ver si mejora o empeora. Si su presión está alta, avísele a su doctor/a.

MULTIMEDIA APPENDIX REFERENCES

1. Hacking D, Haricharan HJ, Brittain K, et al. Hypertension health promotion via text messaging at a community health center in South Africa: a mixed methods study. JMIR Mhealth Uhealth 2016;4(1):e22. PMID:26964505
2. Bobrow K, Farmer AJ, Springer D, et al. Mobile phone text messages to support treatment adherence in adults with high blood pressure (SMS-Text Adherence Support [StAR]): a single-blind, randomized trial. Circulation.2016;133(6):592-600. PMID:26769742
3. Diez-Canseco F, Zavala-Loayza JA, Beratarrechea A, et al. Design and multi-country validation of text messages for an mHealth intervention for primary prevention of progression to hypertension in Latin America. JMIR Mhealth Uhealth 2015;3(1):e19. PMID:25693595
4. Buis LR, Artinian NT, Schwiebert L, Yarandi H, Levy PD. Text messaging to improve hypertension medication adherence in African Americans: BPMED intervention development and study protocol. JMIR Res Protoc 2015;4(1):e1. PMID:25565680
