# Supplementary material for: An Interactive Voice Response and Text Message Intervention to Improve Blood Pressure Control Among Individuals With Hypertension Receiving Care at an Urban Indian Health Organization: Protocol and Baseline Characteristics of a Pragmatic Randomized Controlled Trial
Source: JMIR Res Protoc. 2019 Apr 2;8(4):e11794. doi: 10.2196/11794 (PMC6465973; doi:10.2196/11794)
Supplement: Multimedia Appendix 2 [file resprot_v8i4e11794_app2.pdf]

Multimedia Appendix 2. Baseline survey.

Question	Options	Reference
**About Your Blood Pressure**		
In general, would you say your health is?	1. Excellent<br>2. Very good<br>3. Good<br>4. Fair<br>5. Poor	
Have you ever been told that you had high blood pressure (hypertension)?	1. Yes<br>2. No	
How old were you when you were first told that you had high blood pressure?	[free text response]	
Do you get your blood pressure medicine from the FNCH pharmacy?	1. Yes, I get all of my blood pressure medication from the FNCH pharmacy<br>2. Yes, I get some of my blood pressure medication from the FNCH pharmacy<br>3. No, I do not get my blood pressure medication from the FNCH pharmacy<br>4. No, I do not take any blood pressure medications	
**Home Blood Pressure Monitoring**		
Do you have a blood pressure monitor at home?	1. Yes<br>2. No	
How often do you use your blood pressure monitor at home?	1. Multiple times a day<br>2. Almost daily or daily<br>3. Weekly<br>4. Monthly<br>5. Less than once a month	
Not counting your own home blood pressure monitor, do you ever have your blood pressure checked outside of your doctor's appointments? For example, at the pharmacy or using a friend's blood pressure monitor.	1. Yes<br>2. No	
How often do you have your blood pressure checked outside of your home blood pressure monitor and doctor's appointments? For example, at the pharmacy or using a friend's blood pressure monitor.	1. Multiple times a day<br>2. Almost daily or daily<br>3. Weekly<br>4. Monthly<br>5. Less than once a month	

**In order for high blood pressure (hypertension) medication to work, people have to take it as prescribed. For one reason or another, many people can't or don't always take all of their medicine as prescribed. We want to know how often you have missed your high blood pressure pills. Over the last 7 days…**	All questions below with the following options:<br>1. Never<br>2. Rarely<br>3. Sometimes<br>4. Often<br>5. Always	Voils [1]
…I took all doses of my blood pressure medicine	See above	
… I missed or skipped at least one dose of my blood pressure medicine	See above	
… I was not able to take all of my blood pressure medicine	See above	
**People miss doses for various reasons. Please tell us which reasons contributed to you missing a dose of your blood pressure (hypertension) medicine.**		
I missed my dose because… I was busy	See above	
I missed my dose because… I forgot	See above	
I missed my dose because… The medicine caused some side effects	See above	
I missed my dose because… I worried about taking them for the rest of my life	See above	
I missed my dose because… They cost a lot of money	See above	
I missed my dose because… I came home late	See above	
I missed my dose because… I did not have any symptoms of high blood pressure	See above	
I missed my dose because… I was with friends or family members	See above	
I missed my dose because… I was in a public place	See above	
I missed my dose because… I was afraid of becoming dependent on them	See above	
I missed my dose because… I was afraid they may affect my sexual performance	See above	
I missed my dose because… The time to take them was between my meals	See above	
I missed my dose because… I felt I did not need them	See above	
I missed my dose because... I was traveling	See above	

I missed my dose because… I was supposed to take them too many times a day	See above	
I missed my dose because… I had other medicine to take	See above	
I missed my dose because… They make me need to urinate too often	See above	
I missed my dose because… I ran out of my medicine	See above	
I missed my dose because… I was afraid the medicine would interact with other medicine I take	See above	
I missed my dose because… My blood pressure was too low	See above	
I missed my dose because… I was feeling too ill to take them	See above	
**Other health problems can sometimes get in the way of living healthier lives. Below is a list of common health conditions or illnesses. Please indicate in the first column if you CURRENTLY have the condition. If you do have the condition, please indicate whether or not you receive medicines or some other type of treatment for the condition, and whether the condition limits any of your activities.**	Each stem has options for:<br>1. Do you currently have the condition? (Check if yes)<br>2. Do you receive treatment for it? (Check if yes)<br>3. Does it limit your activities? (Check if yes)	
Prediabetes	See above	
Diabetes	See above	
Heart Disease	See above	
Lung Disease	See above	
Ulcer or stomach disease	See above	
Kidney disease	See above	
Anemia or other blood disease	See above	
Cancer	See above	
Depression	See above	
Osteoarthritis, degenerative arthritis	See above	
Back pain	See above	
Rheumatoid arthritis	See above	
Other medical problems	See above	
**Has a doctor or other health professional ever told you that you...**	1. Yes<br>2. No<br>3. Don't Know	
Had congestive heart failure?	See above	
Had coronary heart disease?	See above	
Had a heart attack (also called a myocardial infarction)?	See above	
Had a stroke?	See above	

**These questions relate to how well you can take care of your health. Please select the answer that best describes how you feel.**	1. Strongly agree<br>2. Agree<br>3. Neither agree nor disagree<br>4. Disagree<br>5. Strongly disagree	Smith [2]
I can take of myself well when it comes to my health	See above	
No matter how I try, my health just doesn't turn out the way I would like	See above	
It is hard for me to find good solutions to my health problems	See above	
I success in the programs I start to improve my health	See above	
I'm generally able to meet my goals when it comes to my health	See above	
My attempts to change things about my health don't work	See above	
Typically, my plans for my health work out well	See above	
I am able to do things for my health as well as most other people	See above	
**OVER THE PAST TWO WEEKS, how often have you been bothered by any of the following problems?**	1. Not at all<br>2. Several days<br>3. More than half the days<br>4. Nearly every day	Lowe [3]
Little interest or pleasure in doing things	See above	
Feeling down, depressed, or hopeless	See above	
**Sometimes experiences in your life can influence your health...**	1. Never<br>2. Once or twice<br>3. A few times<br>4. Many times<br>5. All the time	Adapted from [4-6]
Thinking about your entire life, including your school years, your work life and your personal life... How often do you think you have experienced bias or discrimination because of your race/ethnicity?	See above	
Thinking about your entire life, including your school years, your work life and your personal life... How often do you think members of your family have experienced bias or discrimination because of their race/ethnicity?	See above	
Now consider your lifetime experience with doctors and other health care providers…. How often do you think	See above	

the doctors or other health care providers judged you unfairly or treated you poorly because of your race or ethnicity?		
Now consider your lifetime experience with doctors and other health care providers…. How often do you think the doctors or other health care providers judged members of your family unfairly or treated them poorly	See above	
**Health literacy and numeracy**		Adapted from [7]
How often do you have a hard time understanding written information about your health that you get from your clinic? (This might include information from a doctor or nurse).	1. Always<br>2. Often<br>3. Sometimes<br>4. Rarely<br>5. Never	
How confident are you in filling out medical forms by yourself?	1. Extremely<br>2. Quite a bit<br>3. Somewhat<br>4. A little bit<br>5. Not at a	
How often do you prefer that someone (like a family member or someone else) help you read medical materials?	1. Always<br>2. Often<br>3. Sometimes<br>4. Rarely<br>5. Never	
Which of the following numbers represents the lowest risk? For example, which would you most like to hear from a doctor about your risk for a medical condition?	1. 1 in 10 people<br>2. 1 in 100 people<br>3. 1 in 1000 people<br>4. Don't know	
If the chance of getting a health condition is 20 out of 100 people, this would be the same as having what percent (%) chance of getting the condition?	1. 2%<br>2. 20%<br>3. 200%<br>4. Don't know	
A prescription says "Take one tablet by mouth every 6 hours." If you take your first tablet at 7 a.m., when should you take your second tablet?	1. Later in the morning<br>2. 1 p.m.<br>3. Sometime after 1 p.m.<br>4. Whenever I need it<br>5. 7 p.m.	
Normal blood pressure (the "top" or "systolic" number) is between 80 and 140. If your top blood pressure number	1. Yes<br>2. No<br>3. Close enough<br>4. Don't know	

today is 160, is your blood pressure normal?		
**About Exercise and Diet**		Adapted from Lloyd-Jones [8]
How much time per week do you spend doing moderate exercise (walking, gardening, etc.)?	1. None<br>2. 0-30 minutes<br>3. 31-60 minutes<br>4. 61-90 minutes<br>5. 91-120 minutes<br>6. 121-150 minutes<br>7. More than 150 minutes	
How much time per week do you spend doing vigorous exercise (swimming, running, etc.)?	1. None<br>2. 0-15 minutes<br>3. 16-30 minutes<br>4. 31-45 minutes<br>5. 46-60 minutes<br>6. 61-75 minutes<br>7. More than 75 minutes	
How many servings of fruit or vegetables do you eat in a day?<br>[Include fresh, cooked, frozen, canned, dried, or juices. 1 serving = 1 large orange; 1 small apple; 8 large strawberries; 1/2 cup raisins; 1 large bell pepper; 1 medium potato; 1 cup cooked greens; 2 cups raw greens; 12 baby carrots or 2 medium carrots]	1. 0<br>2. 1<br>3. 2<br>4. 3<br>5. 4<br>6. 4.5 or more	
How many servings of whole grain do you eat in a day?<br>[1 serving = 1 slice whole-wheat or rye bread; 1 cup 100% whole-grain cold cereal; 1/2 cup 100% whole-grain hot cereal (whole oats or oatmeal); 1/2 cup cooked whole-wheat pasta, brown rice, or other grain like barley (about half the size of a baseball); 3 cups popped popcorn]	1. 0<br>2. 1<br>3. 2<br>4. 3 or more	
How many servings of fish do you eat in a week?<br>[1 serving = 3.5 ounces, approximately the same size as a deck of cards]	1. 0<br>2. 1<br>3. 2<br>4. 3 or more	
How many beverages with added sugar do you drink in a week?<br>[1 beverage = 12 ounces]	1. 0<br>2. 1<br>3. 2	

	4. 3<br>5. 4 or more	
Please select all that apply.	1. I avoid eating prepackaged and processed food.<br>2. I rare eat out, but when I do, I seek out lower sodium (salt) options.<br>3. I avoid salt when I'm cooking at home.	
**These questions are about your use of alcohol, tobacco, and other drugs in the past year.**		
How often do you have a drink containing alcohol?	1. Never<br>2. Monthly or less<br>3. 2-4 times per month<br>4. 2-3 times per week<br>5. 4 or more times per week	
How many drinks containing alcohol do you have on a typical day when you are drinking?<br>[1 drink = 12 oz. can of beer, 5 oz. glass of wine, or 1.5 oz. shot of hard liquor]	[free text response]	
Do you use any kind of tobacco, including cigarettes, cigars, a pipe, snuff, or chewing tobacco?	1. Yes<br>2. Yes, but only for ceremonial purposes<br>3. No, I quit within the last year<br>4. No, I quit over a year ago<br>5. No, I have never used tobacco	
In the past year, how often have you used an illegal drug or a prescription medication for non-medical reasons? (Do not include medical or recreational marijuana use)	1. Never<br>2. Sometimes<br>3. Often<br>4. Very often	
**About you**		
How would you describe your gender?	1. Male<br>2. Female<br>3. Other	
Are you Hispanic or Latino/Latina?	1. Yes<br>2. No<br>3. I choose not to answer this question	
Which race(s) are you? Check all that apply.	1. Asian<br>2. Pacific Islander<br>3. American Indian/Alaska Native<br>4. Native Hawaiian<br>5. Black/African American<br>6. White<br>7. Other	

	8. I choose not to answer this question	
If you are American Indian/Alaska Native- What is your Tribal affiliation (which Tribe or Tribes do you most closely identify with)?	[free text response]	
What language are you most comfortable speaking?	1. English<br>2. Spanish<br>3. Other language	
Are you?	1. Married<br>2. Currently living with someone in a marriage-like relationship<br>3. Separated or divorced<br>4. Widowed<br>5. Never married	
What is the highest grade in school you completed?	1. 8th grade or less<br>2. Some high school, but did not graduate<br>3. High school graduate or GED<br>4. Some college, technical/vocational school, or 2 year degree<br>5. 4 year college graduate (B.A., B.S., etc.)<br>6. More than a 4 year college degree	
Are you (choose the one that best describes your situation)?	1. Employed for wages<br>2. Self-employed<br>3. Out of work for 1 year or more<br>4. Out of work for less than 1 year<br>5. A homemaker<br>6. A student<br>7. Retired<br>8. Unable to work	
What is your household annual income from all sources? (If you are unsure or do not know, please give your best guess)	1. Nothing<br>2. Less than $10,000<br>3. $10,000 to $14,999<br>4. $15,000 to $19,999<br>5. $20,000 to $24,999<br>6. $25,000 to $34,999<br>7. $35,000 to $49,999<br>8. $50,000 to $74,999<br>9. More than $75,000<br>10. Prefer not to answer	
**How often do you have?**	1. Always<br>2. Often<br>3. Sometimes<br>4. Almost never<br>5. Never<br>6. Does not apply	
Enough food to eat	See above	
Enough health care	See above	

Enough clothes	See above	
A decent place to live	See above	
Enough money to pay for utility bills (electric, water, gas)	See above	
Adequate childcare	See above	
Enough money to pay for debts and credit card bills	See above	
Has lack of transportation kept you from medical appointments, meetings, work, or from getting things you need for daily living? [Check all that apply]	1. Yes, it has kept me from medical appointments or from getting my medicines<br>2. Yes, it has kept me from non-medical meetings, appointments, work, or from getting things that I needed<br>3. No	
In the last year, have you used SNAP benefits ("food stamps") to purchase food?	1. Yes<br>2. No	

MULTIMEDIA APPENDIX REFERENCES

1. Voils CI, King HA, Neelon B, et al. Characterizing weekly self-reported antihypertensive medication nonadherence across repeated occasions. Patient Prefer Adherence 2014;8:643-650. PMID:24855340
2. Smith MS, Wallston KA, Smith CA. The development and validation of the Perceived Health Competence Scale. Health Educ Res 1995;10(1):51-64. PMID:10150421
3. Lowe B, Kroenke K, Grafe K. Detecting and monitoring depression with a two-item questionnaire (PHQ-2). J Psychosom Res 2005;58(2):163-171. PMID:15820844
4. Kressin NR, Raymond KL, Manze M. Perceptions of race/ethnicity-based discrimination: a review of measures and evaluation of their usefulness for the health care setting. J Health Care Poor Underserved 2008;19(3):697-730. PMID:18677066
5. Brondolo EB, Kelly KP, Coakley V, et al. The Perceived Ethnic Discrimination Questionnaire: development and preliminary validation of a community version. Journal of Applied Social Psychology 2005;35(2):335-365. doi:10.1111/j.1559-1816.2005.tb02124.x
6. Brondolo E, Beatty DL, Cubbin C, et al. Sociodemographic variations in self-reported racism in a community sample of Blacks and Latino(a)s. Journal of Applied Social Psychology 2009;39(2):407-429. doi:10.1111/j.1559-1816.2008.00444.x
7. Brega AG, Jiang L, Beals J, et al. Special diabetes program for Indians: reliability and validity of brief measures of print literacy and numeracy. Ethn Dis 2012;22(2):207-214. PMID:22764644
8. Lloyd-Jones DM, Hong Y, Labarthe D, et al. Defining and setting national goals for cardiovascular health promotion and disease reduction: the American Heart Association's strategic impact goal through 2020 and beyond. Circulation 2010;121(4):586-613. PMID:20089546
