# Supplementary material for: An Interactive Voice Response and Text Message Intervention to Improve Blood Pressure Control Among Individuals With Hypertension Receiving Care at an Urban Indian Health Organization: Protocol and Baseline Characteristics of a Pragmatic Randomized Controlled Trial
Source: JMIR Res Protoc. 2019 Apr 2;8(4):e11794. doi: 10.2196/11794 (PMC6465973; doi:10.2196/11794)
Supplement: Multimedia Appendix 3 [file resprot_v8i4e11794_app3.pdf]

Multimedia Appendix 3. Diagnosis codes for hypertension, diabetes, cardiovascular disease, and depression.

Condition	ICD-9 codes	ICD-10 codes
Hypertension	401-405	I10-I13, I15
Diabetes	250.x, 357.2, 362.0, 366.41	E08.x, E09.x, E10.x, E11.x, E13
Cardiovascular disease	410.x, 411.x, 412.x, 413.x, 414.x, 429.2	I20.0, I20.1, I20.8, I20.9, I21.01, I21.02, I21.09, I21.11, I21.19, I21.21, I21.29, I21.3, I21.4, I22.0, I22.1, I22.2, I22.8, I22.9, I23.0, I23.1, I23.2, I23.3, I23.6, I23.7, I23.8I, I24.0, I24.1, I24.8, I24.9, I25.10, I25.110, I25.111, I25.118, I25.119, I25.2, I25.3, I25.41, I25.42, I25.5, I25.6, I25.700, I25.701, I25.708, I25.709, I25.710, I25.711, I25.718, I25.719, I25.720, I25.721, I25.728, I25.729, I25.730, I25.731, I25.738, I25.739, I25.750, I25.751, I25.758, I25.759, I25.760, I25.761, I25.768, I25.769, I25.790, I25.791, I25.798, I25.799, I25.810, I25.811, I25.812, I25.790, I25.791, I24.798, I25.799, I25.82, I25.83, I25.84, I25.89, I25.9, I51.0
Depression	296.2, 296.3, 296.82, 298.0, 300.4, 301.12, 309.0, 309.1, 309.28, 311.0	F32.0, F32.1, F32.2, F32.3, F32.4, F32.5, F32.8, F32.9, F33.0, F33.1, F33.2, F33.3, F33.40, F33.41, F33.42, F33.9, F34.1, F43.21, F43.23
