# Supplementary material for: An Interactive Voice Response and Text Message Intervention to Improve Blood Pressure Control Among Individuals With Hypertension Receiving Care at an Urban Indian Health Organization: Protocol and Baseline Characteristics of a Pragmatic Randomized Controlled Trial
Source: JMIR Res Protoc. 2019 Apr 2;8(4):e11794. doi: 10.2196/11794 (PMC6465973; doi:10.2196/11794)
Supplement: Multimedia Appendix 4 [file resprot_v8i4e11794_app4.pdf]

Multimedia Appendix 4. Recruitment Diagram.

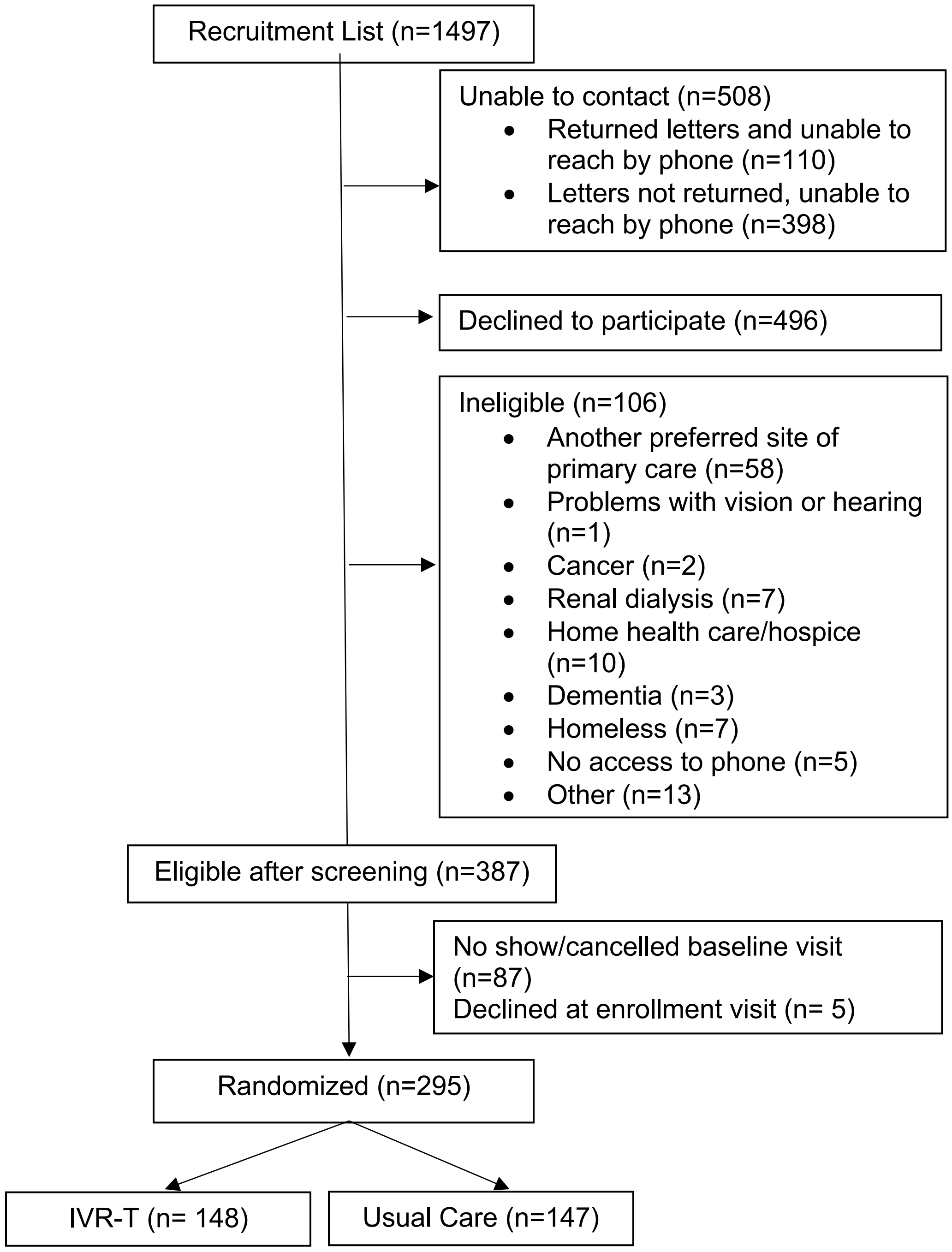

Footnote: An additional 75 individuals were included in the original recruitment pool who had not had a qualifying medical visit at FNCH within the previous 2 years. Among those, 6 were randomized, 4 to the IVR-T group and 2 to the usual care group. These individuals have been excluded from this figure and all subsequent analyses
