# Supplementary figures and images for: An Interactive Voice Response and Text Message Intervention to Improve Blood Pressure Control Among Individuals With Hypertension Receiving Care at an Urban Indian Health Organization: Protocol and Baseline Characteristics of a Pragmatic Randomized Controlled Trial
Source: JMIR Res Protoc. 2019 Apr 2;8(4):e11794. doi: 10.2196/11794 (PMC6465973; doi:10.2196/11794)

Multimedia Appendix 5. Recruitment plot.

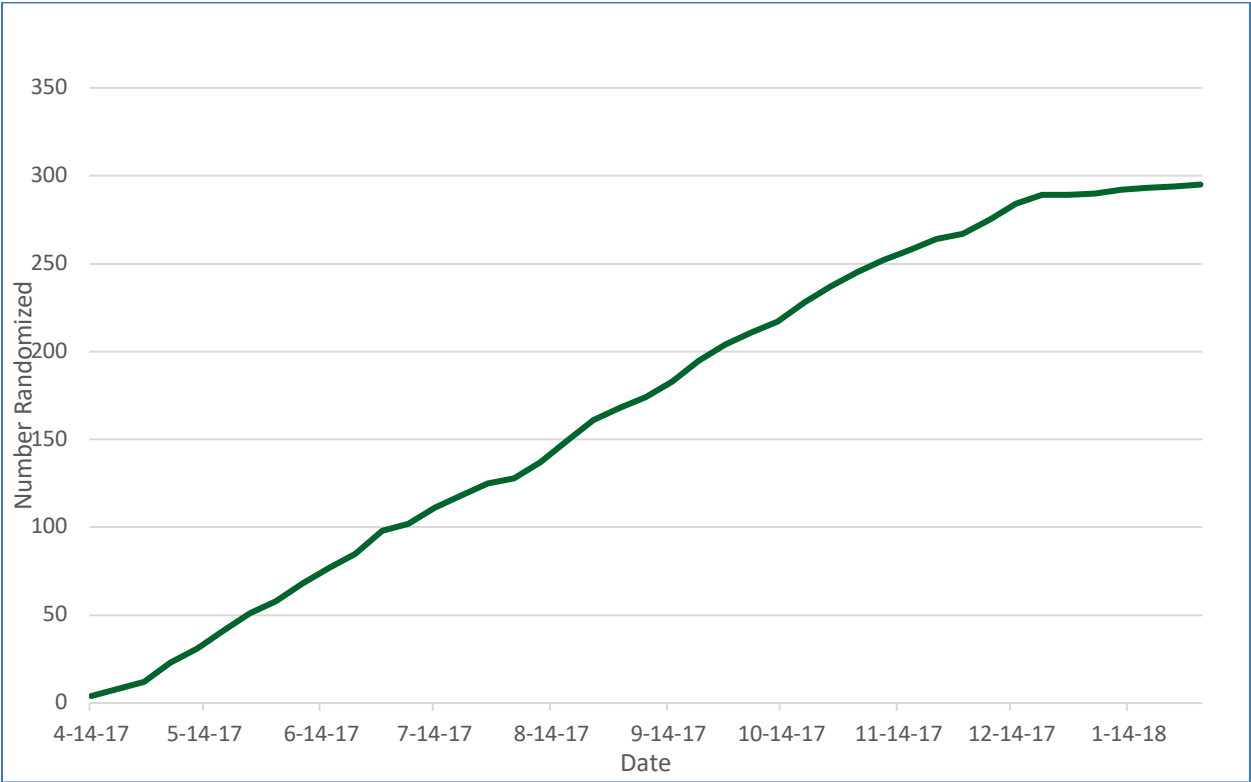

Supplement: Multimedia Appendix 5 [file resprot_v8i4e11794_app5.pdf]
